# *HSD3B* and Gene-Gene Interactions in a Pathway-Based Analysis of Genetic Susceptibility to Bladder Cancer

**DOI:** 10.1371/journal.pone.0051301

**Published:** 2012-12-19

**Authors:** Angeline S. Andrew, Ting Hu, Jian Gu, Jiang Gui, Yuanqing Ye, Carmen J. Marsit, Karl T. Kelsey, Alan R. Schned, Sam A. Tanyos, Eben M. Pendleton, Rebecca A. Mason, Elaine V. Morlock, Michael S. Zens, Zhongze Li, Jason H. Moore, Xifeng Wu, Margaret R. Karagas

**Affiliations:** 1 Norris Cotton Cancer Center, Geisel School of Medicine at Dartmouth, Lebanon, New Hampshire, United States of America; 2 MD Anderson Cancer Center, University of Texas, Houston, Texas, United States of America; 3 Departments of Epidemiology and Pathology and Laboratory Medicine, Brown University, Providence, Rhode Island, United States of America; Duke University Medical Center, United States of America

## Abstract

Bladder cancer is the 4^th^ most common cancer among men in the U.S. We analyzed variant genotypes hypothesized to modify major biological processes involved in bladder carcinogenesis, including hormone regulation, apoptosis, DNA repair, immune surveillance, metabolism, proliferation, and telomere maintenance. Logistic regression was used to assess the relationship between genetic variation affecting these processes and susceptibility in 563 genotyped urothelial cell carcinoma cases and 863 controls enrolled in a case–control study of incident bladder cancer conducted in New Hampshire, U.S. We evaluated gene–gene interactions using Multifactor Dimensionality Reduction (MDR) and Statistical Epistasis Network analysis. The 3′UTR flanking variant form of the hormone regulation gene *HSD3B2* was associated with increased bladder cancer risk in the New Hampshire population (adjusted OR 1.85 95%CI 1.31–2.62). This finding was successfully replicated in the Texas Bladder Cancer Study with 957 controls, 497 cases (adjusted OR 3.66 95%CI 1.06–12.63). The effect of this prevalent SNP was stronger among males (OR 2.13 95%CI 1.40–3.25) than females (OR 1.56 95%CI 0.83–2.95), (SNP-gender interaction *P* = 0.048). We also identified a SNP-SNP interaction between T-cell activation related genes *GATA3* and *CD81* (interaction *P* = 0.0003). The fact that bladder cancer incidence is 3–4 times higher in males suggests the involvement of hormone levels. This biologic process-based analysis suggests candidate susceptibility markers and supports the theory that disrupted hormone regulation plays a role in bladder carcinogenesis.

## Introduction

Bladder cancer is the 4^th^ most common cancer among men in the U.S and accounts for 3% of the cancer deaths [1]. Current methods for detecting bladder tumors involve non-invasive screening by hematauria testing, urine cytology, and bladder imaging, followed by definitive diagnosis using invasive cystoscopy and biopsy. Case-control studies provide evidence of a familial predisposition to bladder cancer [2], [3], [4] indicating that some susceptibility factors may be heritable. Screening programs are more likely to be effective among high-risk subsets of the population, since the prevalence of the disease is higher. Identifying these genetic markers of high-risk groups will enable development of effective screening programs to detect bladder tumors at an early stage.

A number of different mechanisms have adapted to respond to specific types of exogenous damage or aberrant cellular behavior to prevent or repair damage to macromolecules, and to remove damaged cells. We hypothesize that bladder cancer incidence may be affected by genetic variation in each of these major carcinogenesis mechanisms. For example, cell proliferation is controlled by proteins that maintain the synchrony of cell growth, DNA synthesis, mitosis, and cell division [5], [6]. The cell cycle checkpoint response is a signal transduction cascade triggered in response to detection of DNA damage [7]. These signals block cell-cycle progression, giving the cell a chance to repair the lesions before they become permanently integrated into the DNA as mutations. If the damage is too great, the process of apoptosis, or programmed cell death is activated to remove the cells before they proliferate [8], [9], [10]. Genetic variations in critical apoptotic regulators can therefore increase cancer risk. A complex system of DNA repair enzymes provides mechanisms for lesion recognition, protein recruitment, damaged base removal and repair. Genetic variation in DNA repair pathways has well documented effects on bladder cancer risk [11].

The fact that bladder cancer incidence is 3–4 times higher in males suggests that risk may be modified by hormone levels [12]. The excess risk among males is not explained fully by occupational exposures or higher smoking rates. Treatment of animals with androgenic hormones promotes development of more bladder tumors than treatment with estrogenic hormones. The lower risk of bladder cancer among parous compared to nulliparous women also suggests a hormonal axis to the disease [13].

The immune system performs both surveillance for invading pathogenic microorganisms and targeted killing of dysregulated native cells, including tumor cells. Natural killer cells and T-cells can mediate this protective anti-tumor response [14]. Alternatively, the growing tumor may be perceived as a wound, eliciting an inflammatory response that can actually promote tumor development [15]. We hypothesize that genetic variation in molecules involved in immune response and cytokine signaling may modulate cancer susceptibility.

Cellular metabolism and processing of exogenous and endogenous molecules is also integrally related to carcinogenesis. Cells can prevent damage from reactive oxygen species by up-regulating antioxidant enzymes such as glutathione. Metabolic enzymes process xenobiotics, forming biologically reactive intermediates, such as the DNA damaging tobacco byproduct benzo-a-pyrene diol epoxide (BPDE) [16]. In order to divide quickly, tumor cells need to generate large amounts of ATP, which can be accomplished by switching from oxidative phosphorylation to glycolysis [17]. We previously reported that a SNP in the metabolism related gene methylene tetrahydrofolate dehydrogenase (*MTHFD2_01*) was associated with an increased risk of bladder cancer (OR 1.7) [18].

We now test the hypothesis that genetic variation in major processes dysregulated in carcinogenesis: apoptosis, DNA repair, hormone, immune surveillance, metabolism, proliferation, neuronal, telomere, and transport may modify bladder cancer susceptibility. We performed separate analyses of hypothesized cancer-related SNPs by carcinogenesis process, as well as assessed interactions using a large, population-based U.S. case-control study of bladder cancer. Genetic risk variants could be useful for elucidating mechanisms and identifying high-risk groups for targeted screening programs; whereas, interactions with environmental exposures could help identify subgroups of the population who carry the greatest risk.

## Materials and Methods

### Study group

We identified all cases of bladder cancer among New Hampshire residents, ages 25 to 74 years, diagnosed from July 1, 1994 to December 31, 2001 from the State Cancer Registry. Detailed methods have been described previously [19]. Briefly, we interviewed a total of 857 bladder cancer cases, which was 85% of the cases confirmed to be eligible for the study. Slides and blocks were re-reviewed by the study pathologist to assess tumor histology, stage and grade. The stage assigned by the pathologist was used for tumors <stage 2A, while Cancer Registry data on stage was used for higher stage. Analyses were restricted to the participants with a self-reported European ancestry (>95% of subjects in this population), interviewed within two years of primary diagnosis, who were diagnosed with urothelial-cell carcinoma of the bladder of known tumor stage, which were deemed cancerous by histopathology re-review (∼90%), or the original diagnosis if the pathology materials were unavailable, resulting in a case group of 783 participants.

Controls less than 65 years of age were selected using population lists obtained from the New Hampshire Department of Transportation. Controls 65 years of age and older were chosen from data files provided by the Centers for Medicare & Medicaid Services (CMS) of New Hampshire. For efficiency, we shared a control group with a study of non-melanoma skin cancer in New Hampshire covering an overlapping diagnostic period of July 1, 1993 to June 30, 1995 [19]. We selected additional controls for bladder cancer cases diagnosed from July 1, 1995 to June 30, 1998 frequency matched to these cases on age (25–34, 35–44, 45–54, 55–64, 65–69, 70–74 years) and gender. We interviewed a total 1191 controls (the total shared control group and additional controls), which was 70% of the controls confirmed to be eligible for the study. As with cases, analyses were restricted to those who had a self-reported European ancestry (>95% of the controls) resulting in a control group of 1167 participants.

### Personal interview

Informed written consent was obtained from each participant and all procedures and study materials were approved by the Committee for the Protection of Human Subjects at Dartmouth College. Consenting participants underwent a detailed in-person interview, usually at their home. Recruitment procedures for both the shared controls from the non-melanoma skin cancer and additional controls were identical and ongoing concomitantly with the case interviews. Case-control status and the main objectives of the study were not disclosed to the interviewers. To ensure consistent quality of the study interviewer, interviews were tape recorded with the consent of the participants and routinely monitored by the interviewer supervisor. To assess comparability of cases and controls, we asked subjects if they currently held a driver's license or a Medicare enrollment card. Subjects were asked to provide a blood sample. Samples were maintained at 4°C and sent via courier to the study laboratory at Dartmouth within 24 hours for processing and analysis.

### Genotyping

DNA was isolated using Qiagen genomic DNA extraction kits (QIAGEN Inc., Valencia, CA) from peripheral circulating blood lymphocyte specimens harvested at the time of interview or subsequently from an outside laboratory. Genotyping was performed on all DNA samples of sufficient concentration (563 urothelial cell carcinoma cases, 863 controls), using the GoldenGate Assay system Illumina, Inc., San Diego, CA). We genotyped a total of 1,522 SNPs, mostly from the Illumina Cancer Panel (1,421 SNPs in approximately 400 hypothesized cancer-related genes). SNPs were selected within the coding, intronic and flanking regions hypothesized to be potentially functional of the genes of interest, including a median of 3 SNPs per gene. A total of 24 (<2%) of SNPs were excluded from the analysis due to minor allele frequency <5% or a low genotype call rate. The SNPs reported had call rates of ≥99.2%. Samples repeated on multiple GoldenGate plates yielded the same call for 99.9% of SNPs and 99.5% of samples submitted were successfully genotyped.

The main goal of the study was to assess the relationship between genetic variation in nine major carcinogenesis processes and bladder cancer susceptibility. These nine processes are: Apoptosis, DNA Repair, Immune, Hormone, Metabolism, Neural, Proliferation, Telomere, and Transport/Signaling. We grouped SNPs that were involved in each process according to the Database for Annotation, Visualization, and Integrated Discovery (DAVID) Gene Ontology (GO) search engine (http://www.geneontology.org/GO.tools.microarray.shtml) [20]. The number of SNPs analyzed by functional group was as follows: apoptosis n = 135, DNA repair n = 224, immune n = 243, hormone n = 68, metabolism n = 340, neural n = 11, proliferation n = 304, telomere n = 27, transport/signaling n = 170 (Table S1 contains a complete list of these SNPs).

### External Comparison

We compared the results of the *HSD3B2* SNP identified in our New Hampshire study population with those of a proxy SNP that was in linkage disequilibrium (LD = 1.0) with this SNP using data from the Texas Bladder Cancer Study (TXBCS). Cases (n = 497) and controls (n = 957) in the Texas Bladder Cancer Study (TXBCS) who had been genotyped using a Genome-Wide Association panel were derived from an ongoing hospital-based bladder cancer case-control study [21]. Cases were newly diagnosed, histologically confirmed and previously untreated incident bladder cases recruited from the University of Texas MD Anderson Cancer Center and Baylor College of Medicine. Healthy controls were recruited from Kelsey Seybold, the largest multispecialty, managed-care physician group in the Houston metropolitan area. Controls were individuals with no prior history of cancer (except non-melanoma skin cancer) frequency-matched to cases by age (±5 years), sex and ethnicity. Informed consent was obtained from all study participants before the collection of epidemiological data and blood samples by trained MD Anderson staff interviewers. The study was approved by the IRB of MD Anderson Cancer Center, Baylor College of Medicine and the Kelsey-Seybold Clinic.

### Statistical analysis

#### Analysis of individual SNPs by group

Individual SNPs were analyzed separately within each of the five biologic pathways of interest using multivariate logistic regression (SAS version 9.1). Within each of the nine major carcinogenesis processes, we tested hypothesized associations between SNPs and bladder cancer susceptibility using logistic regression with adjustment for age, gender, and smoking status. Assuming incomplete dominance, we tested heterozygous vs. wildtype; variant vs. wildtype, as well as a dominant model (heterozygous+variant vs. wildtype). Analyses were performed for all urothelial cell carcinoma cases together, as well as stratified into invasive (stage II, III, IV) and non-invasive (stage 0,I) tumors, and by gender. All analyses were adjusted for age, gender, and smoking status (never, former, current). Statistical significances of the interactions were assessed using likelihood ratio tests comparing the models with and without interaction terms.

*P* values represent two-sided statistical tests. To correct for multiple comparisons within each group of SNPs, we also calculated *P*-values adjusted for the False Discovery Rate (FDR) for the total number of SNPs within a functional group and report SNPs with FDR adjusted *P*-values<0.25.

#### Analysis of SNP-SNP Interactions

We used the nonparametric multifactor dimensionality reduction (MDR) approach described in detail elsewhere [22], [23], [24], [25] and reviewed by Moore [26]. MDR is a data reduction (i.e. constructive induction) approach that seeks to identify combinations of multilocus genotypes that are associated with either high risk or low risk of disease. We used an adaptation of the ReliefF algorithm as a pre-filtering step to help identify epistatic or non-additive, interacting SNPs in MDR [27]. We used 10-fold cross-validation and 1000-fold permutation testing. In addition, we used Statistical Epistasis Networks to identify gene-gene interactions within each functional group [28]. We further evaluated the two top-ranked interactions (mutual information weights that exceeded 0.01, permutation testing *P*-values<0.001). We included interaction terms in a logistic regression model, adjusting for age, gender and smoking status. Statistical significances of the interactions were assessed using likelihood ratio tests comparing the models with and without the interaction terms. The primary difference between these two methods of identifying SNP-SNP interactions is that MDR analysis is independent of the genetic model. It assigns each cell of a 3×3 interaction table as either high risk or low risk, regardless of their frequency in the population, and then groups these genotype combinations into a two level variable. In contrast, the Statistical Epistasis Network analysis uses wildtype - wildtype as the referent group. Statistical epistasis networks also separate interactions (depicted as edge width) from main effects (depicted as vertex size).

Statistical significance of individual logistic regression results was considered at *P*<0.001 and we restricted our report to SNPs that meet this cutoff. To avoid reporting unstable estimates, we conservatively set the minimum cell size to 20 heterozygous or variant cases and controls.

## Results

Cases and controls in our study were comparable in age. The genotyped participants had similar characteristics as the overall study participants (Table 1). A higher percentage of bladder cancer cases were males than controls (Table 1). More cases than controls reported that they were current smokers. As our study is population-based, the majority (83%) of our cases were non-invasive at initial diagnosis.

**Table 1:** New Hampshire study population characteristics.

	Overall Controls				Genotyped Controls			
			TCC Cases				TCC Cases	
	N	(%)	N	(%)	N	(%)	N	(%)
**Gender**								
female	457	(39.2)	188	(24.0)	327	(37.9)	135	(24.0)
male	710	(60.8)	595	(76.0)	536	(62.1)	428	(76.0)
**Total**	1167		783		863		563	
**Reference age**								
<40	55	(4.7)	22	(2.8)	41	(4.8)	17	(3.0)
40–49	143	(12.3)	58	(7.4)	86	(10.0)	37	(6.6)
50–59	226	(19.4)	190	(24.3)	168	(19.5)	130	(23.1)
60–69	467	(40.0)	324	(41.4)	356	(41.3)	242	(43.0)
> = 70	276	(23.7)	189	(24.1)	212	(24.6)	137	(24.3)
**Smoking status**								
Never	392	(33.6)	131	(16.7)	292	(33.8)	98	(17.4)
Former	566	(48.5)	385	(49.2)	432	(50.1)	273	(48.5)
Current	201	(17.2)	254	(32.4)	139	(16.1)	191	(33.9)
**Stage**								
Non-invasive	-		659	(84.2)	-		476	(84.5)
Invasive	-		79	(10.1)	-		55	(9.8)
Tis	-		45	(5.7)	-		32	(5.7)
**Recurrence**								
no	-		337	(43.0)	-		248	(44.1)
yes	-		389	(49.7)	-		280	(49.7)
**Deceased**								
no	-		466	(59.5)	-		333	(59.2)
yes	-		317	(40.5)	-		230	(40.9)

missing: smoking n = 21, recurrence n = 57.

Variants for a SNP in the hormone regulation gene 3-beta-hydroxysteroid dehydrogenase type II (*HSD3B2*) had an increased risk of bladder cancer (overall OR 1.94 95%CI 1.36–2.75) compared to those who were wildtype (Table 2). The overall findings for SNP HSD3B2_23 remained significant when analysis was restricted to non-invasive tumors. The effect was stronger for men (variant OR 2.13 95%CI 1.40–3.25) than women (variant OR 1.56 95%CI 0.83–2.95), with a statistically significant SNP-gender interaction (*P*-interaction 0.048).

**Table 2:** HSD3B2 SNP and bladder cancer risk in the New Hampshire population, by gender.

		N controls (%)	N cases (%)	OR (95%CI)	*P*-value	FDR	OR (95%CI)	OR (95%CI)	*P-inter-action*
				Overall			Male	Female	
HSD3B2_23	GG	606 (70.2)	377 (67.0)	1.00 (referent)			1.00 (referent)	1.00 (referent)	
g.10174G>A	GA	182 (21.1)	100 (17.8)	0.95 (0.71–1.27)	0.726	0.964	1.13 (0.81–1.59)	0.60 (0.34–1.05)	
rs1417608	AA	75 (8.7)	86 (15.3)	1.94 (1.36–2.75)	<0.001	<0.001	2.13 (1.40–3.25)	1.56 (0.83–2.95)	
	GA/AA	257 (29.8)	186 (33.1)	1.25 (0.98–1.57)	0.070	0.077	1.42 (1.06–1.90)	0.82 (0.51–1.32)	0.055

			Invasive						
	GG	606 (70.2)	36 (65.5)	1.00 (referent)			1.00 (referent)	1.00 (referent)	
	GA	182 (21.1)	13 (23.6)	1.32 (0.68–2.58)	0.417	0.842	1.5 (0.71–3.34)	0.81 (0.21–3.12)	
	AA	75 (8.7)	6 (10.9)	1.45 (0.58–3.65)	0.424	0.842	1.45 (0.48–4.44)	1.37 (0.26–7.07)	
	GA/AA	257 (29.8)	19 (34.5)	1.36 (0.76–2.44)	0.305	0.324	1.52 (0.76–3.01)	0.96 (0.30–2.99)	0.495

			Non-invasive						
	GG	606 (70.2)	341 (67.1)	1.00 (referent)			1.00 (referent)	1.00 (referent)	
	GA	182 (21.1)	87 (17.1)	0.90 (0.67–1.22)	0.511	0.980	1.08 (0.76–1.53)	0.57 (0.31–1.03)	
	AA	75 (8.7)	80 (15.8)	1.99 (1.39–2.84)	<0.001	<0.001	2.22 (1.45–3.40)	1.46 (0.75–2.84)	
	GA/AA	257 (29.8)	167 (32.9)	1.22 (0.96–1.57)	0.106	0.114	1.42 (1.06–1.90)	0.82 (0.51–1.32)	0.048

Adjusted for age, gender, smoking.

We then validated this analysis using cases and controls from the Texas Bladder Cancer Study. We identified a proxy SNP that was in linkage disequilibrium (LD = 1.0) with the risk-associated SNP that we first identified in the New Hampshire population. We observed a 3-fold increase in bladder cancer risk associated with the variant genotype (OR 3.66 95%CI 1.06–12.63) compared with wildtype (Table 3).

**Table 3:** HSD3B2 proxy SNP is associated with bladder cancer risk in the Texas population.

		N controls (%)	N cases (%)	OR (95%CI)	*P*-value	OR (95%CI)	OR (95%CI)
				Noninvasive		Male	Female
HSD3B2	TT	792 (82.8)	422 (84.9)	1.00 (referent)		1.00 (referent)	1.00 (referent)
g.119981049T>C	TC	161 (16.8)	68 (13.7)	0.78 (0.57–1.07)	0.121	0.75 (0.53–1.07)	0.92 (0.46–1.85)
rs1341015	CC	4 (0.42)	7 (1.4)	3.66 (1.06–12.63)	0.041	3.15 (0.88–11.31)	-

Adjusted for age, gender, smoking.

SNPs analyzed in relation to overall bladder cancer risk that we reported previously (e.g. in metabolism SNPs methylenetetrahydrofolate dehydrogenase (NADP+ dependent) 2 (*MTHFD2*), alcohol dehydrogenase 1C (*ADH1C*); telomerase SNPs telomerase-associated protein 1 (*TEP1*) and telomerase reverse transcriptase (*TERT*)) also met the current criteria for statistical significance, false discovery rate, and minimum sample size per cell that we set for this restricted analysis of urothelial cell carcinoma risk and showed similar trends for risk of both non-invasive and invasive tumors [18].

We used Multifactor Dimensionality Reduction (MDR) algorithms to detect the top potential interactions between the analyzed SNPs in relation to urothelial cell carcinoma risk [27]. MDR analysis of the Immune-related genes yielded the top ranking SNP combination: GATA3_23, and CD81_04 (testing balance accuracy 0.56, cross-validation consistency 3/10). This interaction was statistically significant by the likelihood ratio test, with a reduced bladder cancer risk associated with the heterozygous/variant genotype of these SNPs vs. wildtype (*P* = 0.0003) (Table 4). The best MDR two-way models for the other processes did not have a likelihood ratio test *P* values that met our criteria for statistical significance after adjustment for age, gender and smoking status (data not shown).

**Table 4:** SNP-SNP interactions identified by Multifactor Dimensionality Reduction.

SNP a		SNP b		N	(%)	N	(%)	Odds Ratio (95%CI)	*P* interact
GATA3_23	TT	CD81_04	GG	689	(82.2)	488	(85.2)	1.0 (ref)	
g.IVS3+2491C>T	CT/CC	g.-1784A>G	GA/AA	149	(17.8)	85	(14.8)	0.41 (0.25–0.66)	0.0003
rs569421		rs708155							

missing GATA3_23 n = 2, CD81_04 n = 39.

 In addition, we ran Statistical Epistasis Network models to test pairwise interactions between SNPs, as described [28]. Logistic regression analysis of the top ranked SNP pairs (information gain >0.011) was then performed with adjustment for age, gender and smoking status. Network graphs depict the top SNP combinations, as ranked by permutation *P*-value and interaction strength, for the DNA repair, Immune and Metabolism groups (Figure S1). Individuals with the variant forms of DNA repair genes postmeiotic segregation increased 1 (*PMS1*) and nibrin (*NBS1*) had a 4-fold higher risk of bladder cancer (OR 4.15 95%CI 1.79–9.66) compared to those wildtype for these SNPs (Table 5). In contrast, reduced risks were observed for Immune–related SNPs in interferon regulatory factor 3 (*IRF3*) and interleukin 6 (*IL-6*) (OR 0.39 95%CI 0.24–0.62), and for Metabolism SNP combinations of catechol-O-methyltransferase (*COMT*) and apolipoprotein B (*APOB*) (OR 0.35 95%CI 0.21–0.58), and for prostaglandin-endoperoxide synthase 2 (*PTGS2*) and ATP-binding cassette, sub-family C, member 4 (*ABCC4*) (0.23 95%CI 0.11–0.49). Other potential interactions shown in the network graphs did not meet our likelihood ratio test criteria for statistical significance after adjustment for age, gender, and smoking status. Hardy Weinberg equilibrium *P*-values for the control population were (HSD3B2_23 rs1417608 NH = 0.46e-19, rs1341015 TX = 0.38, GATA3_23 = 0.005, CD81_04 = 0.45, PMS1_26 = 0.10, NBS1_01b = 0.66, IRF3_01 = 0.30, IL6_01 = 0.24, COMT_28b = 0.003, APOB_07 = 0.09, PTGS2_05 = 0.79, ABCC4_04 = 0.50).

**Table 5:** SNP-SNP interactions identified by Statistical Epistasis Network Analysis.

SNP a		SNP b		N	(%)	N	(%)	Odds Ratio (95%CI)	*P* interact
**DNA Repair**									
PMS1_26	TT/CT	NBS1_01b	CG/GG	826	(95.8)	530	(93.8)	1.0 (ref)	
rs1233258	CC	rs1805794	CC	36	(4.2)	35	(6.2)	4.15 (1.79–9.66)	0.0009
g.IVS5-1656C>T		g.Ex5-32C>G p.E185Q							
**Immune**									
IRF3_01	AA/GG	IL6_01	CC/GG	698	(59.8)	492	(62.6)	1.0 (ref)	
rs2304204	AG	rs1800795	CG	165	(14.1)	71	(9.0)	0.39 (0.24–0.617)	<0.0001
g.-924A>G		g.Ex2C>G							
**Metabolism**									
COMT_28b	AA/GG	APOB_07	CT/TT	619	(53.0)	436	(55.5)	1.0 (ref)	
rs6518591	AG	rs1800481	CC	234	(20.1)	122	(15.5)	0.35 (0.21–0.58)	<0.0001
g.IVS2+1485T>C		g.-392C>T							
									
PTGS2_05	AA/GG	ABCC4_04	CC/TT	788	(67.5)	516	(65.7)	1.0 (ref)	
rs689466	AG	rs3765535	CT	68	(5.8)	44	(5.6)	0.23 (0.11–0.485)	0.0001
g.Ex2A>G		g.IVS17-65T>C							

We tested for evidence of population stratification using the entire set of 1,421 SNPs analyzed in this study. A Q-Q plot comparing the observed to the expected *P*-value distribution suggests that population stratification is negligible for this study (Figure S2). We calculated the genome inflation factor (λ) to be 1.05 (95%CI 0.92–1.08) [29].

## Discussion

We observed that genetic variation in the 3′UTR flanking region of the hormone regulation gene 3-beta-hydroxysteroid dehydrogenase type 2 (*HSD3B2*) was associated with nearly a two-fold increase in bladder cancer risk. This SNP is relatively prevalent in the population, with variant alleles detected in more than 30% of our case group. *HSD3B* encodes NAD+ dependent microsomal enzymes that catalyze biosynthesis and inactivation of many types of steroid hormones. They catalyze the formation of dihydrotestosterone and dihydroprogesterone [30] (Figure S3a). In the prostate, they inactivate dihydrotestosterone, which binds and stimulates gene transcription via androgen-responsive elements [31]. Several studies have previously associated *HSD3B* polymorphisms with increased prostate cancer risk and aggressiveness [31], [32], [33], [34]. Specifically, two *HSD3B2* SNPs in the 3′UTR (rs1819698 and rs1538989) were associated with significantly increased risk of prostate cancer in people of European descent (OR 2.66 95%CI 1.13–6.24) [31]. These prostate cancer related SNPs are in high linkage disequilibrium with our bladder cancer associated flanking SNP, rs1417608 (r^2^ 0.841 and 1.0, respectively). SNP rs1538989 affected the binding of transcriptional activator PU.1 in an allele-specific manner [31]. SNP rs1819698 is also computationally predicted to disrupt microRNA binding sites for miR-3658 (DeltaS 0.571). Our risk associated SNP (rs1417608) is also in linkage disequilibrium with another miR binding site disrupting 3′UTR SNP, rs1361530 (r^2^ 0.818), which may affect binding of miR-423-5p (DeltaS 0.62), miR-1914* (DeltaS 0.591), and miR-3658 (DeltaS 0.571) (http://tare.medisin.ntnu.no/mirsnpscore). These computationally predicted relationships are speculative and functional characterization will be necessary to verify disruption of miRNA binding experimentally.

The *HSD3B2* SNP that we found associated was not included on the large genome-wide association panels used for the recent studies of bladder cancer [21], [35], [36]. A search for SNPs in linkage disequilibrium revealed other flanking 3′UTR SNPs that were included on the Illumina SNP panels analyzed in these studies (rs1341015, rs11805965, rs10923825). Analysis of the proxy SNP rs1341015 in the Texas Bladder Cancer Study population affirmed a 3-fold higher risk. The association may not have been reported previously due to the strong association among men, and in non-invasive cancers; whereas, prior genome-wide scan studies focused on main effects in populations, and some had a larger proportion of invasive and high risk tumors [21], [35], [36]. The predominance of bladder cancer in males relative to females raises the possibility of a role for dysregulated hormone levels in the etiology of this disease. In women, parity is related to a lower risk for bladder cancer compared with nulliparity, also supporting the hypothesis that hormones may modify bladder cancer risk [37], [38]. Recent animal studies further implicate a specific role for androgens and the androgen receptor in bladder cancer. Specifically, androgen and androgen receptor depletion protected mice from development of chemical carcinogen induced bladder tumors. Dihydrotestosterone supplementation partially restored tumor formation to androgen receptor knockout mice [12].

In the Immune-response pathway, we observed an interaction between SNPs in *GATA3* and *CD81*, such that the combined heterozygous variant genotypes were associated with reduced bladder cancer risk. CD81 promotes T-cell activation, proliferation, and IL-2 production [39] (Figure S3b). GATA3 is a transcription factor that binds and activates T-cell receptors alpha and delta, among numerous other targets such as interleukins and interferon gamma (Figure S3c). Over-expression of GATA3 protein was observed in 67% of urothelial carcinomas [40]. GATA3 polymorphisms have been associated with reduced breast cancer risk [41]. The risk-modifying effect of these immune-related SNPs reinforces the importance of immune-response, inflammation, or infection in the etiology of bladder cancer. Bladder is one of the few cancers with robust responses to immunotherapy. The inflammatory response to Bacillus Calmette-Guerin (BCG) instillation in the bladder results in an anti-tumorigenic effect involving neutrophils [42]. Furthermore, we and others consistently observe lower bladder cancer risk among users of non-steroidal anti-inflammatory drugs (NSAIDs) [43].

As with the immune-response SNPs, several of the other interactions that we observed conferred a lower risk of bladder cancer associated with a combination of heterozygous genotypes. This effect is biologically plausible since a moderate modification of effect can be beneficial, while more extreme effects that might be conferred by homozygous variant genotypes could be harmful. An alternative explanation for identification of interactions among heterozygotes is a statistical power issue whereby the combination of two homozygous variant genotypes was too infrequent in the population to be able to detect interactions. Functional analyses of these genotypic combinations would be needed to help elucidate these effects.

The population-based nature of this study is an advantage for the potential generalizability of the results. Limitations include small numbers of highly invasive lesions that restrict our ability evaluate the impact of genotype on this subset of aggressive cancers. Nevertheless, our data suggest novel SNP-SNP and SNP-gender interactions that modify bladder cancer risk. In particular, a prevalent SNP flanking the hormone regulatory gene *HSD3B* was associated with a greater than two-fold increased risk among males. This finding was successfully confirmed by a proxy SNP in the Texas Bladder Cancer Study population and further validation will ensure the applicability of these potential genetic markers prior to clinical use. This observation adds support to the theory that hormone dysregulation may be important in the etiology of bladder cancer. Identifying hormone dysregulation as a new causal factor for bladder cancer could lead to novel prevention strategies in the future. Genetic susceptibility genes can be used as markers for high-risk subpopulations who would benefit from screening strategies to detect bladder tumors at early stages. Susceptibility markers can also be used to guide the setting of limits on DNA damaging agents in the environment and workplace that are protective of more sensitive individuals.

## Supporting Information

Figure S1 Statistical Epistasis Networks showing gene-gene interactions in relation to bladder cancer risk. The strengths of main effect and pair-wise interaction effect were measured using entropy-based mutual information and information gain associated with each SNP and each pair-wise combination of SNPs. SNP genotypes were modeled according to increasing variant allele dose (0 = wildtype, 1 = heterozygous, 2 = homozygous variant). Results are depicted in network diagrams. Permutation testing (1000 fold) was used to calculate the statistical significance of each pair of SNPs. Each node represents a SNP and its size denotes its main effect. The width of an edge linking two SNPs denotes their pair-wise interaction strength. Graphs presented represent the top ranked interactions that have a permutation testing *P*<0.02: **S1a.** DNA repair network, **S1b.** Immune network, **S1c.** Metabolism network.
(JPG)

Figure S2 Q-Q plot of *P*-values. As a test for population stratification, we compared the observed distribution of *P*-values from the logistic regression analysis (y-axis) to that expected under the null distribution (x-axis).
(JPG)

Figure S3 Downstream regulatory relationships for HSD3B1, CD81 and GATA3. Pathway Studio software (Ariadne Genomics) was used to depict known downstream gene functions based on references from the published literature. Nodes represent genes, proteins, small molecules, or cellular processes. Arrows represent the type of relationship between nodes. **S3a.** HSD3B2 (solid black – chemical reaction, green- molecular synthesis, grey- molecular), **S3b.** CD81 (blue – expression, dark grey – direct regulation), **S3c.** GATA3 (green – promoter binding).
(JPG)

Table S1 Full list of genotyped SNPs organized by functional group.
(XLSX)
