# Supplementary figures and images for: HSD3B and Gene-Gene Interactions in a Pathway-Based Analysis of Genetic Susceptibility to Bladder Cancer
Source: PLoS One. 2012 Dec 19;7(12):e51301. doi: 10.1371/journal.pone.0051301 (PMC3526593; doi:10.1371/journal.pone.0051301)

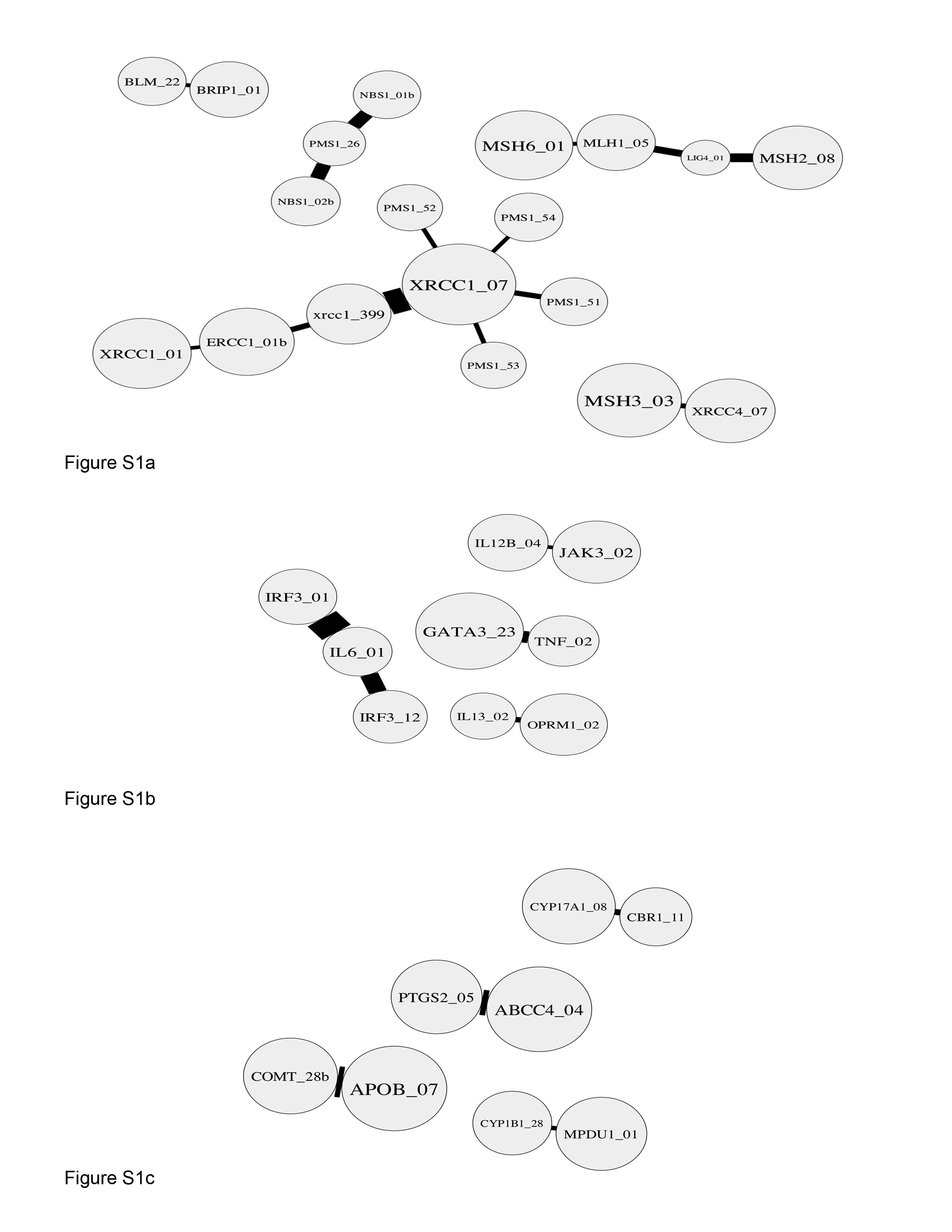

Supplement: Figure S1 — Statistical Epistasis Networks showing gene-gene interactions in relation to bladder cancer risk. The strengths of main effect and pair-wise interaction effect were measured using entropy-based mutual information and information gain associated with each SNP and each pair-wise combination of SNPs. SNP genotypes were modeled according to increasing variant allele dose (0 = wildtype, 1 = heterozygous, 2 = homozygous variant). Results are depicted in network diagrams. Permutation testing (1000 fold) was used to calculate the statistical significance of each pair of SNPs. Each node represents a SNP and its size denotes its main effect. The width of an edge linking two SNPs denotes their pair-wise interaction strength. Graphs presented represent the top ranked interactions that have a permutation testing P<0.02: S1a. DNA repair network, S1b. Immune network, S1c. Metabolism network. (JPG) [file pone.0051301.s001.jpg]

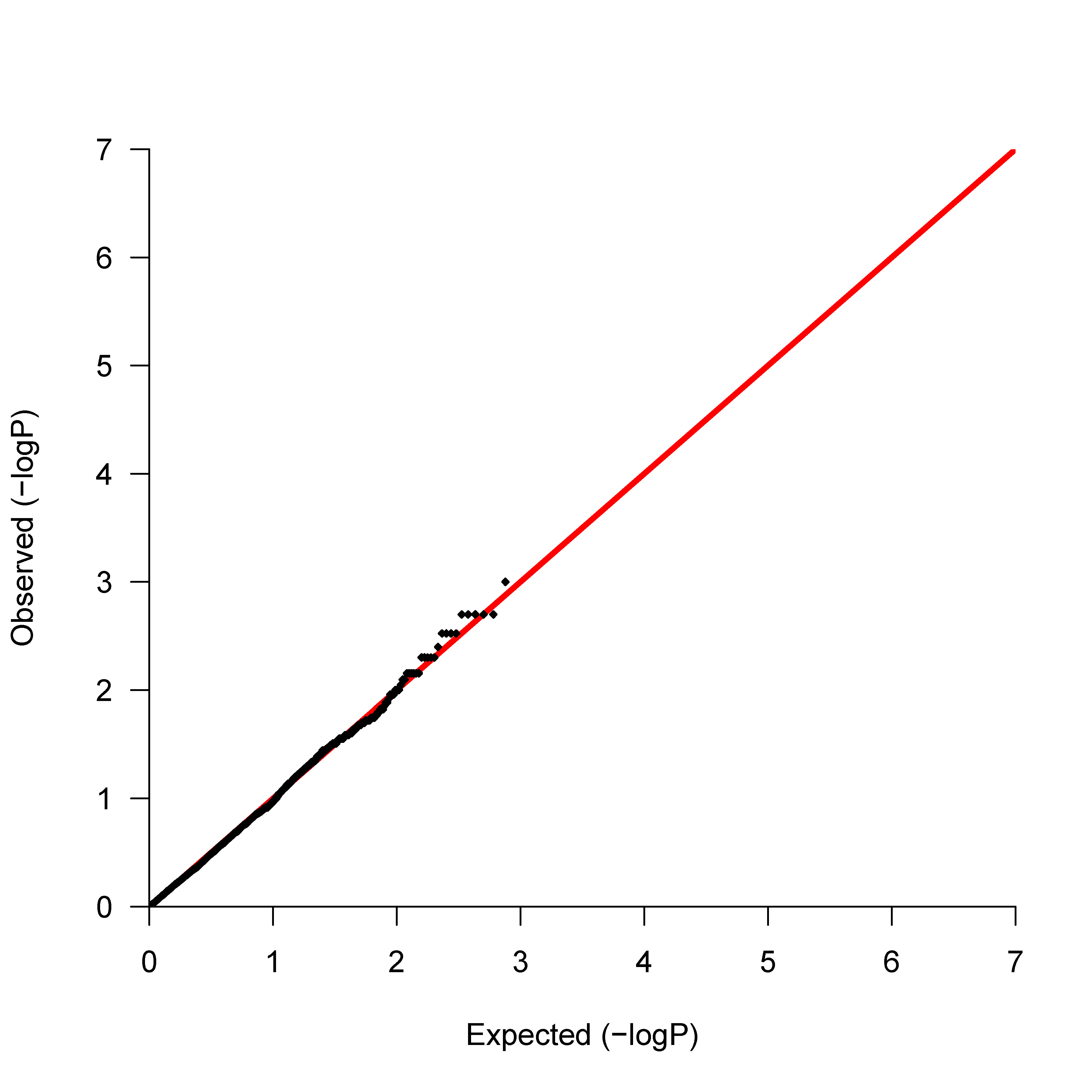

Supplement: Figure S2 — Q-Q plot of P-values. As a test for population stratification, we compared the observed distribution of P-values from the logistic regression analysis (y-axis) to that expected under the null distribution (x-axis). (JPG) [file pone.0051301.s002.jpg]

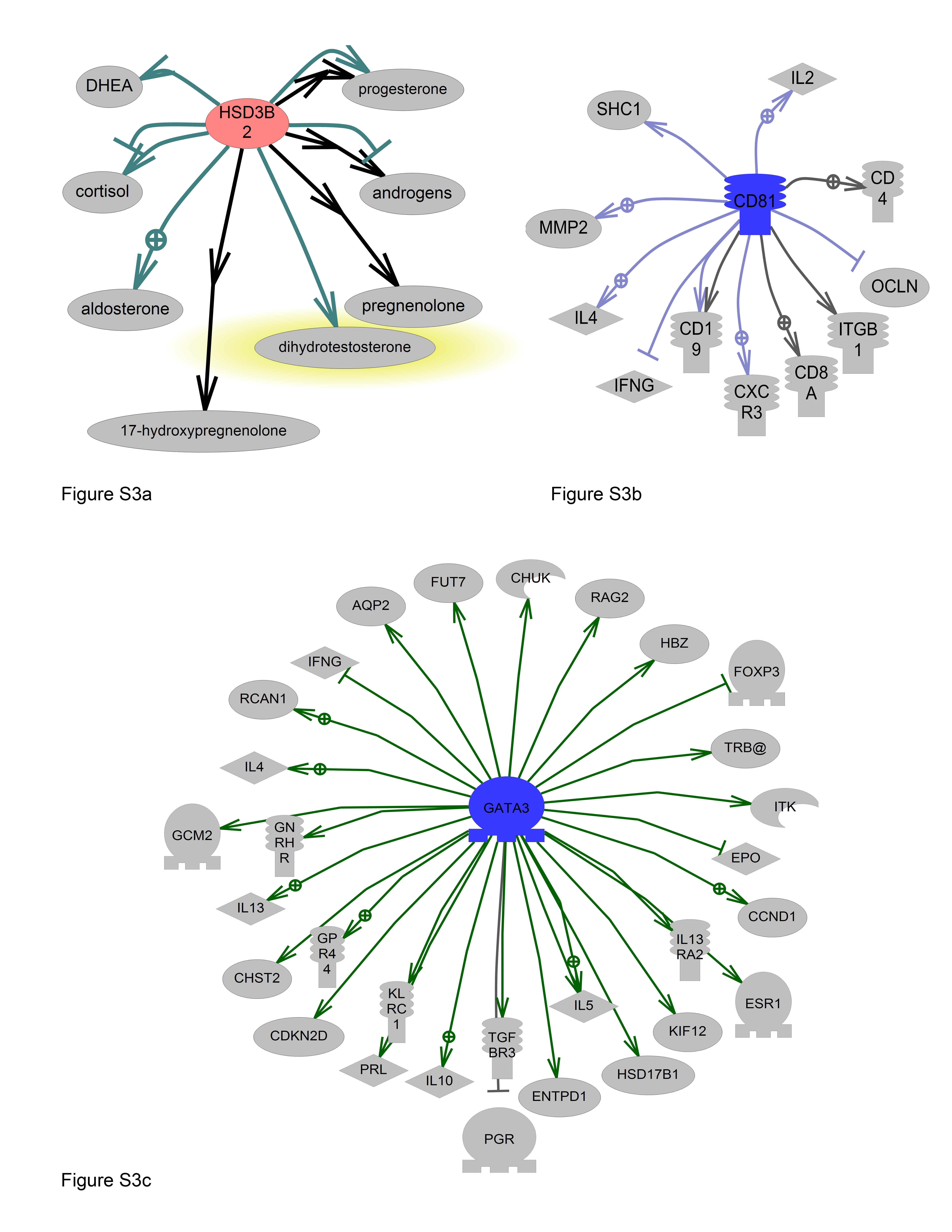

Supplement: Figure S3 — Downstream regulatory relationships for HSD3B1, CD81 and GATA3. Pathway Studio software (Ariadne Genomics) was used to depict known downstream gene functions based on references from the published literature. Nodes represent genes, proteins, small molecules, or cellular processes. Arrows represent the type of relationship between nodes. S3a. HSD3B2 (solid black – chemical reaction, green- molecular synthesis, grey- molecular), S3b. CD81 (blue – expression, dark grey – direct regulation), S3c. GATA3 (green – promoter binding). (JPG) [file pone.0051301.s003.jpg]
